# Use and management of traditional medicinal plants by Maale and Ari ethnic communities in southern Ethiopia

**DOI:** 10.1186/1746-4269-10-46

**Published:** 2014-06-04

**Authors:** Berhane Kidane, Tinde van Andel, Laurentius Josephus Gerardus van der Maesen, Zemede Asfaw

**Affiliations:** 1 Ethiopian Institute of Agricultural Research, Forestry Research Center, P.O. Box 58532, Addis Ababa, Ethiopia; 2 Biosystematics Group, Wageningen University and Research Center, P.O. Box 647, 6700 Wageningen, AP, The Netherlands; 3 Naturalis Biodiversity Center, Leiden University, P.O. Box 9517, 2300 Leiden, RA, The Netherlands; 4 Department of Plant Biology and Biodiversity Management, The National Herbarium, Addis Ababa University, P.O. Box 3434, Addis Ababa, Ethiopia

## Abstract

**Background:**

Around 80% of the people of Ethiopia are estimated to be relying on medicinal plants for the treatment of different types of human health problems. The purpose of this study was to describe and analyse the use and management of medicinal plants used for the treatment of human health problems by the Maale and Ari communities in southern Ethiopia.

**Methods:**

Quantitative and qualitative ethnobotanical field inquiries and analytical methods including individual and focus group discussions (18), observations, individual interviews (n = 74), preference ranking and paired comparison were used. Data were collected in three study sites and from two markets; the latter surveyed every 15 days from February 2011 to February 2012.

**Results:**

A total of 128 medicinal plant species, belonging to 111 genera and 49 families, used as herbal medicine by Maale and Ari communities were documented. Predominantly harvested plant parts were leaves, which are known to have relatively low impact on medicinal plant resources. Species with high familiarity indices included *Solanum dasyphyllum, Indigofera spicata, Ruta chalepensis, Plumbago zeylanica and Meyna tetraphylla*. Low Jaccards similarity indices (≤ 0.33) indicated little correspondence in medicinal plant use among sites and between ethnic communities. The dominant ways of medicinal plant knowledge acquisition and transfer is vertical: from parents to children through oral means. Gender and site significantly influenced the number of human medicinal plants known currently in the study sites. Age was only a factor of significance in Maale. Marketing of medicinal plants harvested from wild and semi-wild stands is not common. Expansion of agricultural land and lack of cultivation efforts by local communities are mentioned by locals to affect the availability of medicinal plant resources.

**Conclusion:**

*S. dasyphyllum, I. spicata, P. zeylanica, M. tetraphylla,* and *Oxalis radicosa* need to be considered for phytochemical and pharmacological testing to verify their efficacy and determine their dosages. Land use planning and development initiatives in the area and beyond need to sharply focus on strategies that could alleviate the major threats affecting medicinal plant resources in the landscape and encourage their cultivation to enhance their availability and complement ex-and in-situ conservation.

## Background

Ethiopia is a country with regional differences in access to health services [1]. It is estimated that traditional medicine (about 95% herbal) is used by 80% of the Ethiopian population for the treatment of different types of human health problems [2]. Medicinal plants are used as a major source for health promotion, prevention and cure [3,4]. The traditional use of medicinal plants by most Ethiopians in health care system is generally ascribed to the incomplete coverage of the modern medical system, unaffordable and not always available prescription drugs [5-7] and the widespread belief in the effectiveness of herbal medicine [8,9].

Medicinal plant knowledge is shaped by the ecological diversity of the country [10], known to be site-specific [11] and varies across peoples with different religious, linguistic and cultural backgrounds [7]. In Ethiopia, there are over 70 ethnic communities, residing in different ecological regions [12] and the studies so far have shown extensive medicinal plant knowledge, acquired through centuries of experience. Although several studies have been conducted on medicinal plants throughout the country e.g. [13-18]**,** the full wealth of this knowledge has not yet been sufficiently studied. We therefore document medicinal plants used by the Maale and Ari communities less studied Ethiopian communities and evaluate similarities and differences among sites and between the two communities.

Ethiopian farmers’ knowledge on medicinal plants may be influenced by certain demographic characteristics. Awas [11] and Giday et al. [16] showed that gender and age significantly affected farmers’ knowledge on traditional medicine. However, in other farming communities, gender and age had no significant effect on useful plant knowledge [19]. In order to effectively preserve indigenous knowledge, we need to find out whether socio-demographic factors (age, gender, religion, educational level, family size) or locality affect the level of medicinal plant knowledge among the Maale and Ari communities.

Traditionally used medicinal plants and associated knowledge are disappearing at an alarming rate [20]. Natural and anthropogenic factors contribute to these losses but threatening factors may vary from one region to the other [17]. Therefore, we want to understand factors that threaten local traditional medicinal plant resources and knowledge, which are important for decision makers for their policy formulation and analysis. Mechanisms of medicinal plant knowledge acquisition and transfer affect knowledge continuity within a community. Most studies conducted in Ethiopia so far have shown that the major mechanism for transfer of ethnomedicinal knowledge is oral [20], although Fassil [8] indicated existence of pharmacopias (ancient written medicinal plant knowledge) in monasteries in the northern highlands of Ethiopia. In order to preserve herbal medicine traditions, we need to reveal the local mechanisms of knowledge transfer.

Marketing of medicinal plants may have implications on natural resources, depending on the species marketed, the type of plant part harvested, volumes sold and cultivation efforts of commercial species. A few of the ethnobotanical studies in Ethiopia attempted to highlight the issue of marketing of medicinal plants [21,16]. However, the studies were of cross-sectional nature and did not conduct systematic repeated visits and data collection and elements that affect the marketing of traditional medicinal plants were hardly addressed. In this paper, we describe and analyse the use and management of medicinal plants by Maale and Ari communities by answering the following research questions: 

1) Which plant species are used as medicines at different sites by the two communities and for what purposes?

2) What are the mechanisms of herbal medicinal plant knowledge transfer among different social groups?

3) Which demographic factors significantly influence medicinal plant knowledge?

4) How do the two communities differ in their use and handling of medicinal plants?

5) What factors threaten medicinal plant resources in the study area?

6) Which medicinal plants are commercialized and what elements affect their marketing?

## Materials and methods

### Study area and selection of informants

**Figure 1:**
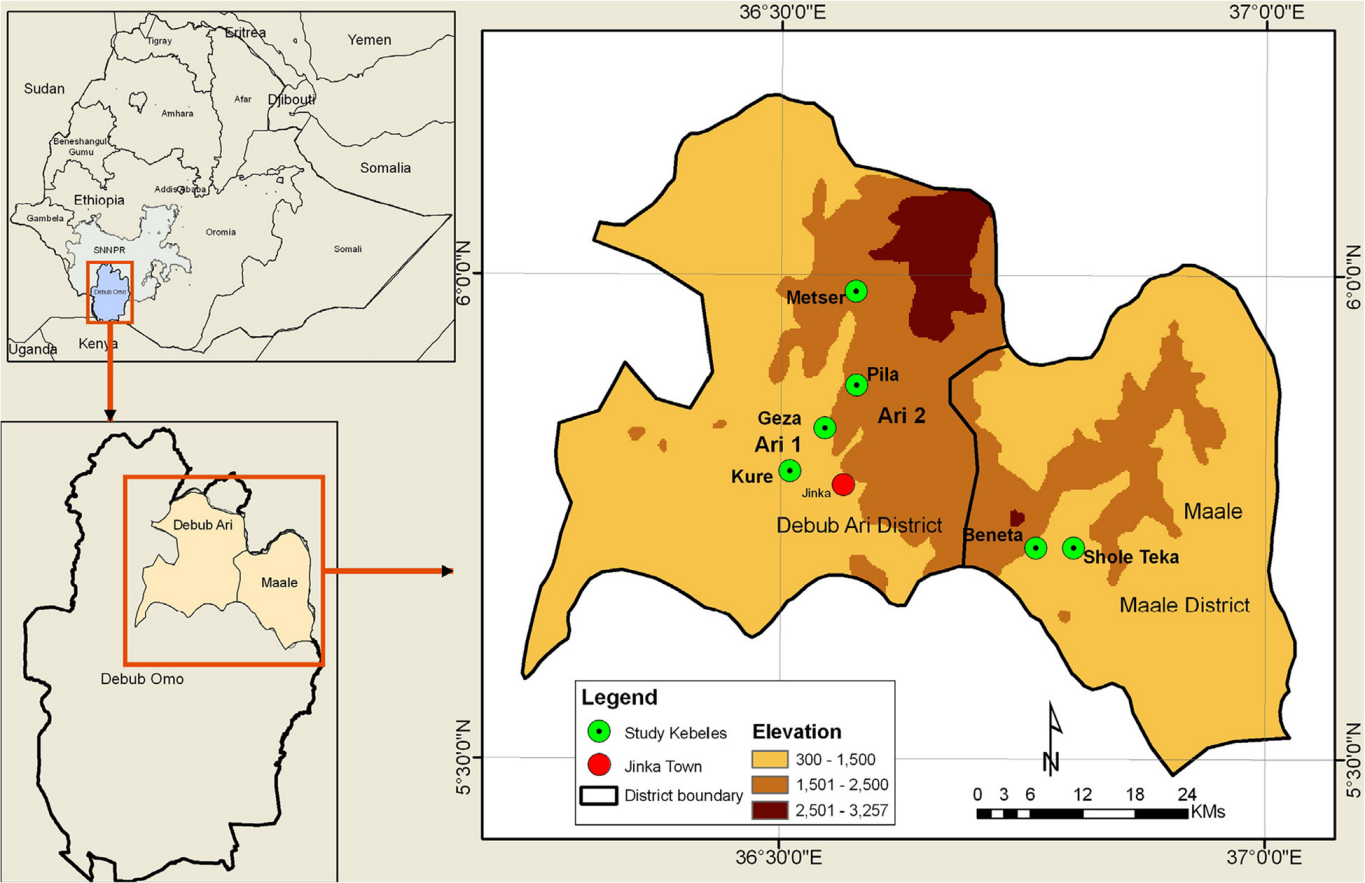
Location map of the study area in southern Ethiopia.

The study was conducted in the Maale and Debub Ari districts of southern Ethiopia, where the Maale and Ari communities reside. The study area is located at about 750 km south of the Ethiopian capital, Addis Ababa.A reconnaissance survey was conducted from July to August 2010 in the two study districts. Well settled areas by the two ethnic communities within the two districts studied. Detailed fieldwork was conducted from August 2010 to October 2012. Prior to detailed data collection, individual and focus group discussions (18) with informants were conducted. At each kebele (the lowest formal administrative unit in rural Ethiopia), 10 to 12 informants from different socio-demographic groups were involved in the focus group discussions. In general we used stratified simple random sampling for the selection of the study sites, study kebeles and study participants. The stratifying variables were ethnic communities and altitude. The three study sites selected for detailed formal survey were Maale, Ari 1 and Ari 2 (Figure 1). For each site, two kebeles (Shole teka and Beneta in Maale, Kure and Geza in Ari 1 and Pilla and Metser in Ari 2) were randomly selected (Figure 1). The Ari 1 and Maale study kebeles were selected from the area with altitude of 500-1500 m.a.s.l. However, the Ari 2 study site is located within altitude ranging from 1500-2500 with mean altitude of 1700 m. Despite the presence of areas with altitude ranging from 1500-2500 in Maale district, the area was not included in the study because there are no substantial settlements.

*Acacia - Commiphora* and *Combretum – Terminalia* woodland is characterizes the remnant vegetation of Maale study site; the mean altitude of the selected study site is 1350 m.a.s.l. The remnant vegetation of Ari 1 is characterized by moist evergreen Afromontane forest and *Combretum – Terminalia* woodland [22] with a mean altitude of 1330 m.a.s.l. The forest area is converted to agricultural land, with scattered trees in the fields and home gardens of Ari 2. The language of Ari is Araf, while the Maale language is spoken by the Maale communities. The 2007 population census shows that there were 84657 in Maale and 212 389 in Debub Ari [23].

A total of 74 study participants (24 from Ari 1, 24 from Ari 2 and 26 from Maale), belonging to the two ethnic communities were considered for the interviews. The age range of randomly selected study participants lied between 20 and 60 except a single participant who was older: 70 years**.** Oral informed consent was obtained from each participant prior to conducting the interviews.

### Ethical approval

Written permission (approval) was obtained from South Omo “zonal” council and also from the Maale and Debub Ari district council offices before the start of the study.

### Consent

Oral and also signed informed consent were obtained from the study participants for the publication of this report and any accompanying images.

### Data collection

We used semi-structured interviews, field observations, preference ranking and paired comparison following the standard ethnobotanical methods and procedures as given by various authors [24-26] to collect information on various aspects of medicinal plants in the two districts. The semi-structured interviews contained questions on common health issues, medicinal plants, plant parts used, preparation and application methods, dosage, and possible threats to medicinal plant resources. Moreover, study participants were asked how they acquired medicinal plant knowledge and whether they were willing to transfer this knowledge. Each informant was visited at least two times during the study period in order to validate the information provided. Following the recommended procedures by Alexiades [25], responses of the study participants that contradicted with each other were not considered for analysis.

Voucher specimens of medicinal plants were collected with the assistance of key informants following standard botanical procedures. Preliminary plant identification was done in the field while collecting and further identification and confirmation was done at the National Herbarium (ETH) of Addis Ababa University (AAU) using taxonomic keys provided in the relevant volumes of the Flora of Ethiopia and Eritrea [27-33]. Finally, the identified specimens were deposited at the ETH. Scientific names were checked for accuracy by means of the Plant List [34].

Between February 2011 and February 2012, surveys were carried out at Beneta market (Maale area) and Jinka market (Debub Ari). Markets were visited every fifteen days and all medicinal plants sold were documented with their price, source and additional trade information.

### Data analysis

Descriptive statistics were used to calculate average numbers of medicinal plants and illness types listed by the study participants, and to quantify acquisition and transfer of traditional knowledge. In Maale, ten key informants were selected to participate in a preference ranking exercise following Martin [24] for eight plants used to treat the most frequently cited health problem (in this case ascariasis). Key informants ranked plant species according to their perceived efficacy: the most effective being given a value of one, and the least effective a value of eight. Priority ranking was used to classify major issues that affected the availability of medicinal plants in the study sites.

The calculated Jaccard’s similarity indices [35] were used to compare similarity of medicinal plant knowledge among the studied communities. This index uses plant presence/positive reply or absence/negative reply data sets and is expressed as:

$$\mathrm{JI}=\frac{c}{a+b+c}$$

Where JI is the Jaccard similarity index, c is the number of species shared by the study sites, a is the number of species in study site A only and b is the number of species in study site B only. The JI values range between 0 and 1, whereby a value of 1 indicates complete similarity.

Familiarity index (FI) was used as an indicator of the popularity of a species [36]. FI was defined as the number of respondents that mentioned a species for a specific use, divided by the total number of respondents. The value of FI varies between 0 and 1, whereby a value of 1 represents the highest familiarity of a medicinal plant in the study site.

$$\mathrm{FI}=\frac{\mathrm{Frequency}\;\mathrm{of}\;a\;\mathrm{given}\;\mathrm{species}\;\mathrm{mentioned}\;\mathrm{as}\;a\;\mathrm{medicine}}{\mathrm{Total}\;\mathrm{number}\;\mathrm{of}\;\mathrm{respondents}}$$

Multiple regression analysis was employed to reveal demographic factors that predicted traditional knowledge [37]. We used the number of plants known as dependent variable and considered age, gender, religion, education level, family size and geographic location as explanatory variables. Variables that were highly correlated (r ≥ 0.9) were not included in the model [37]. Independent t-tests were employed to compare the differences between gender and age groups. All statistical methods were carried out in the program SPSS 20.0.

## Results and discussion

### Medicinal plants reported

A total of 128 medicinal plant species, belonging to 111 genera and 49 families were reported by Maale and Ari communities for the treatment of 48 different types of health problems (see Table 1). The family Lamiaceae was represented by the highest number of species (19) followed by Leguminosae (10 species), Acanthaceae, Solanaceae, Cucurbitaceae, and Malvaceae (each 8 spp) and Rubiaceae and Compositae (6 spp each). The highest number (92 spp) was reported in Maale, while 54 species were jointly documented in Ari 1 and Ari 2, of which 18 species were shared. Medicinal plants were used as the first line of treatment by 96%, 83%, and 88% of the respondents of Maale1, Ari 1 and Ari 2 respectively. This indicates that traditional medicine plays a significant role in the primary health care system of the Maale and Ari communities.

**Table 1:** Medicinal plants used in Maale and Ari communities with plant parts used, growth form and applications

**N**^**o**^	**Vernacular names: Maale (M), Araf (A)**	**Ailments**	**Scientific name**	**Family Name**	**Voucher №**	**Parts used**	**Growth form**	**Application route**	**Study sites**
1	Tsinkaso (M)	Eye disease (infection), Headache	*Barleria ventricosa* Hochst. ex Nees	Acanthaceae	210 , 356	Leaves	Herb	Topical	M
2	Moro Golodo (M)	Gastritis	*Blepharis maderaspatensis* (L.) Roth.	Acanthaceae	234, 637	Whole	Herb	Oral	M
3	Golodo (M)	Oral trash, Gastritis, Malnutrition	*Justicia bizuneshiae* Ensermu	Acanthaceae	357	Leaves	Herb	Oral	M
4	Busino (M)	Amoebiasis, Stomach ache, Diarrhea	*Hypoestes forsskaolii (*Vahl) R. Br.	Acanthaceae	30	Root	Herb	Oral	M
5	Kati Murso (M)	Constipation, Ascariasis	*Thunbergia alata* Boj. ex Sims	Acanthaceae	232,366, 391	whole	Climber	Oral	M
6	Majimaylo(M)	Tape worm	*Celosia trigyna* L.	Amaranthaceae	679	Leaves	Herb	Oral	M
7	Tsami shinkurt (A)	Stomach ache	*Allium sativum* L.	Amaryllidaceae	-	Bulb	Herb	Oral	A1, A2
8	Salvano (M)	Ascariasis	*Ozoroa insignis* Delile	Anacardiaceae	177 224 289 302	Stem bark	Tree	Oral	M
9	Kubri (M)	Diarrhea, Toothache, Food poisoning, Vomiting	*Rhus natalensis* Krauss	Anacardiaceae	28	Leaves	Shrub	Oral	M
10	Muralatse (M)	Food poisoning, vomiting	*Uvaria leptocladon* Oliv*.*	Annonaceae	24	Leaves	Shrub	Oral	M
11	Afi Deshe (A)	Evil eye	*Agrocharis melanantha* Hochst.	Apiaceae	309	Leaves	Herb		A 2
12	Etsewayo (M), Ountinkam (A)	Gastritis, Headache Evil eye	*Centella asiatica (*L.) Urban	Apiaceae	36,128	whole	Herb	Oral	M, A1, A2
		Swelling	*Centella asiatica* (L.) Urban			Whole	Herb	Topical	
13	Ambelto (M), Almi (A)	Tonsilites	*Carissa spinarum* L.	Apocynaceae	33,119	Leaves	Tree	Oral	M, A1
		Snake protection	*Carissa spinarum* L.			Root	Tree	Smoke	
		Stomach ache,	*Carissa spinarum* L.			Root	Tree	Oral	
		Evil eye	*Carissa spinarum* L.			Root	Tree	Inhale	
14	Pijie (M)	Ascariasis	*Pergularia daemia (*Forssk.) Chiov.	Apocynaceae	304	Root	Climber	Oral	M
		Malnutrition (Child)	*Pergularia daemia* (Forssk.) Chiov.			Leaves		Oral	M
15	Metse (M)	Retained placenta	*Leptadenia hastata* (Pel's.) Decne.	Apocynaceae	101	Leaves	Shrub	Oral	M
		Liver disease(Ara)	*Leptadenia hastata* (Pel's.) Decne.			Leaves		Inhale	
16	Zolpe (M)	Liver disease (Ara)	*Stereospermum kunthianum* Cham.	Bignoniaceae	282,402	Leaves	Tree	Inhale	M
17	Achenti (A)	Stomach ache	*Cynoglossum lanceolatum* Forssk.	Boraginaceae	2	Root	Herb	Oral	A2
18	Kolpo (M)	Common cold	*Brassica carinata* A. Braun	Brassicaceae		Leaves	Herb	Oral	M
19	Feto	Common cold	*Lepidium sativum* L.	Brassicaceae	-	Seed	Herb	Oral	A2
20	Afi Deshe (A)	Evil eye	*Monopsis stellarioides (*Presl) Urb.	Campanulaceae	246	Leaves	Herb	Oral	A2
	Guni deshe (A)	Snake bite	*Monopsis stellarioides* (Presl) Urb.						
21	Chedi (A)	Oral trash	*Garcinia livingstonei* T.Anders.	Clusiaceae	14	Fruit	Tree	Topical	A2
22	Yemdir Berbere (A)	Tooth ache, Tonsillitis	*Acmella caulirhiza* Delile	Compositae	89,313	Flower	Herb	Topical	A1
23	Dunko (M), Duno (A)	Stomach ache	*Artemisia absinthium* L.	Compositae	-	Leaves with stem	Herb	Oral	M, A1
		Evil eye, Sudden disease, Headache	*Artemisia absinthium* L.			Leaves with stem		Oral/Inhale	
24	Hachenti (M)	Diarrhea	*Bidens pilosa* L.	Compositae	233	Root	Herb	Oral	M
25	Azi deshe (A)	Swelling(eti)	*Conyza gouanii* (L.) Willd.	Compositae	311	Leaves	Herb	Topical	A2
26	Rebasho (M) Haro mato (M)	Rheumatism	*Tagetes minuta* L.	Compositae	203	Leaves	Herb	Topical	M
		Amoebiasis	*Tagetes minuta* L.			Leaves	Herb	Oral	
27	Gera (A)	Malaria	*Vernonia amygdalina* Delile	Compositae	-	leaves	shrub	Oral	M, A1
28	Kwakuch deshe (A)	Skin disease (‘Kwakucha)	*Cuscuta campestris* Yuncker	Convolvulaceae	259	Leaves with succulent stem	Herb	Topical	A1
29	Lago (M)	Malnutrition (Child) Diarrhea, Hheart disease, Gastritis	*Ipomoea obscura* (L.) Ker-Gawl.	Convolvulaceae	211	Leaves	Herb/climber type	Oral	M
30	Welanke (M)	Liver disease	*Ipomoea spathulata* Hall.f.	Convolvulaceae	173,404	Leaves	Herb	Inhale	M
31	Kamakarsho (M)	Ascariasis	*Combretum aculeatum* Vent.	Combretaceae	287,504	Leaves	Shrub	Oral	M
32	Gaal (M)	Typoid	*Terminalia brownii* L.	Combretaceae		Leaves	Tree	Oral	M
		Snake bite	*Terminalia brownii* L.			Bark		Oral/Topical	
33	Hachirindo (M)	Lower extremity weakness	*Cucumis dipsaceus* Ehrenb. ex Spach	Cucurbitaceae	15	Leaves	Herb	Topical	M
		Amoebiasis	*Cucumis dipsaceus* Ehrenb. ex Spach			Whole		Oral	
34	Botayilashe (M), Bota (A)	Tape worm	*Cucurbita pepo* L.	Cucurbitaceae	-	Seed	Herb	Oral	M, A1
	Bota	Tape worm	*Cucurbita pepo* L.			Seed	Herb	Oral	
35	Shuntee (M)	Mouth wound	*Kedrostis foetidissima* (Jacq.) Cogn	Cucurbitaceae	84	Leaves	Herb	Topical	M
36	Choko (M)	Amoebiasis	*Momordica foetida* Schumach.	Cucurbitaceae	204, 242	**Whole**	Herb	Oral	M
37	Najie (M)	Evil eye	*Momordica pterocarpa* Hochst. ex A. Rich.	Cucurbitaceae	20	Leaves	Climber	Topical	M
		Amoebiasis	*Momordica pterocarpa* Hochst. ex A. Rich.			Leaves	Climber	Oral	
38	Ounsi (M)	Stomach ache	*Euclea divinorum* Hiern	Ebenaceae	26	Root	Shrub	Oral	M
39	Sauto zao (M)	Stomach bloating, food poisoning, Vomiting	*Acalypha fruticosa* Forssk.	Euphorbiaceae	98	Leaves	Shrub	Oral	M
		Stomach ache	*Acalypha fruticosa* Forssk.			Stem		Oral	M
40	Sauti (M)	Eye injury	*Acalypha volkensii Pax*	Euphorbiaceae	223	Leaves	Herb	Topical	M
41	No local name	Eye disease (Cataract)	*Euphorbia hirta L.*	Euphorbiaceae	496	Latex	Herb	Topical	M
42	Tsedo (M)	Rabies	*Euphorbia tirucalli* L.	Euphorbiaceae	285	Root	Shrub	Oral	M
43	Tsamo desho (M)	Wound	*Ricinus communis* L.	Euphorbiaceae	350	Seed	Shrub	Topical	M,A2
44	Beto (M) Beta (A)	Gonorrhea ,Tape worm	*Croton macrostachyus* Hochst. ex Ferret & Galinier	Euphorbiaceae		Leaf bud	Tree	Oral	M, A1, A2
45	Gaina deshe/Azi deshe (A)	Stomach bloating,	*Geranium arabicum* Forssk.	Geraniaceae	312,445	Leaves	Herb	Oral	A2
		Swelling	*Geranium arabicum* Forssk.			Leaves	Herb	Topical	
46	Bato ketero deshe (M)	Amoebiasis	*Pelargonium quinquelobatum* Hochst. ex A.Rich.,	Geraniaceae	227	Whole	Herb	Oral	M
47	Afi Deshe (A)	Evil eye	*Hypericum peplidifolium* A.Rich*.*	Hypericaceae	263	Leaves	Herb	Oral	A1,A2
48	Harsi deshe/ Gaina deshe/Shai Shar (A)	Diarrhea	*Ajuga leucantha* Lukhoba	Lamiaceae	274, 278, 441	Leaves	Herb	Oral	A1,A2
49	Baye Apo Desho (M)	Stomach ache, Heart, Rheumatism	*Becium filamentosum* (Forssk.) Chiov.	Lamiaceae	196,221,324	Whole	Herb	Oral	M
50	Bokolo (M), Dumfeken (A)	Stomach bloating, vomiting	*Clerodendrum myricoides* (Hochst.) Vatke	Lamiaceae	195,346	Leaves	Shrub	Oral	M, A1, A2
		Evil eye	Clerodendrum myricoides *(Hochst.) Vatke*	Lamiaceae	38	Root	Shrub	Oral ,Inhale	
51	Apo Desho (M)	Evil eye	*Endostemon tereticaulis* (Poir.) M.Ashby	Lamiaceae	99,165	Whole	Herb	Topical	M
52	Tsamo desho (M)	Cold	*Fuerstia africana* T.C.E. Fr.	Lamiaceae	230	Leaves	Herb	Topical	M
53	Pelo Tsala (M)	Stomach ache, Amoebiasis, Stomach bloating, Head ache, Food poisoning, Vomiting	*Leucas abyssinica* (Benth.) Briq.	Lamiaceae	167,321	Leaves	Shrub	Oral	M
		Rheumantism	*Leucas abyssinica* (Benth.) Briq.			Leaves		Topical	
54	Chergicola	Pus from Ear, nose, mouth; Eye disease (cataract), Rheumatism	*Leucas glabarata (*Vahl) Sm. in Rees	Lamiaceae	237, 281, 320, 365	Leaves	Herb	Topical	M
		Headache				Leaves		Oral/inhale	
55	Azi deshe	Swelling	Leucas martinicensis (Jacq.) R.Br.	Lamiaceae	315	Leaves	Herb	Topical	A2
56	Lamo (M)	Stomach ache	*Ocimum basilicum* L.	Lamiaceae	222 ,317	Leaves	Herb	Oral	M
57	Gurdarindo (M)	Headache, Diarrhea Stomach bloating, Stomach ache, Vomiting	*Ocimum forskolei* Benth.	Lamiaceae	214 , 235	Whole	Herb	Oral	M
58	Pasi kedo	Heart disease	*Ocimum laliifolium* Hochst. ex Benth.	Lamiaceae	225,37	Leaves	Herb	Oral	M,
	Demakesse	Headache	*Ocimum lamiifolium* Hochst. ex Benth.		134	Leaves		Oral/Inhale	A1, A2
59	Kuliti kup (M)	Herpes simplex ( “Mich”)	*Ocimum urticifolium* Roth	Lamiaceae	133	Leaves	Shrub	Topical	A1,A2
		Stomach ache, Vomiting	*Ocimum urticifolium* Roth					Oral	
		Headache	*Ocimum urticifolium* Roth					Inhale	
60	Pero (M)	Amoebiasis, Stomach ache	*Plectranthus barbatus* Andrews	Lamiaceae	85	Root	Herb	Oral	M
61	Dumio (M)	Lower extremity weakness	*Plectranthus cylindraceus* Hochst. ex Benth.	Lamiaceae	229,503	Leaves	Herb	Topical	M
62	Ketero Desho Solelo (M)	Stomach ache, Amoebiasis, Diarrhea	*Plectranthus longipes* Baker	Lamiaceae	464	whole	Herb	Oral	M
63	Banjirindo (M)	Ease of Birth	*Plectranthus punctatus* (L.f) L.'Hér.	Lamiaceae	207,318,369	Whole	Herb	Oral	M
64	Anchip (M)	Amoebiasis, Diarrhea, Stomach bloating, Stomach ache, Food poisoning	*Pycnostachys abyssinica* Fresen.	Lamiaceae	205, 319	Leaves	Herb	Oral	M
65	Sheto (M)	Constipation	*Satureja abyssinica* (Benth.) Briq.	Lamiaceae	292, 690	Leaves	Herb	Oral	M
66	Zene gaime Deshe (A)	Stomach ache	*Satureja paradoxa* (Vatke) Engl. ex Seybold	Lamiaceae	310, 371	Leaves	Herb	Oral	A2
67	Kayneka/Digita (A)	Stomach ache	*Calpurnia aurea* (Ait.) Benth.	Leguminosae	247,272	Leaves	shrub	Oral	A1, A2
		Diarrhea	*Calpurnia aurea* (Ait.) Benth*.*			Root	Herb	Oral	
68	Aro Dor deshe (M)	Malnutrition (Child)	*Chamaecrista mimosoides* (L.) Greene	Leguminosae	197,322	Whole	Herb	Oral	M
69	Dongordoso (M)	Tonsilites	*Indigofera spicata* Forssk.	Leguminosae	202, 251, 256,261, 345,388	Root	Herb	Oral	M, A1
	Afi Deshe/Gaina Deshe/Wesfat deshe (A)	Diarrhea, Evil eye Ascariasis, Stomach ache	*Indigofera spicata* Forssk.		467	Whole		Oral	
70	Birbira (A)	To close wound caused by Jiggers	*Millettia ferruginea* (Hochst.) Baker	Leguminosae	-	Seed	Tree	Topical	A2
71	Dawrake (M)	Liver disease	*Piliostigma thonningii* (Schumach.) Milne-Redh.	Leguminosae	117	Leaves	Tree	Inhale	M
72	Ara Deshe (A)	Liver disease (Ara)	*Senna petersiana* (Bolle) Lock	Leguminosae	260	Leaves	Shrub	Inhale	A1, A2
73	Karhaleko (M)	Stomach ache, Diarrhea , Food poisoning, Vomiting, Ascariasis	*Senna singueana (*Delile) Lock	Leguminosae	59,28	Root	Shrub	Oral	M
74	Dino desho (M)	Diarrhea ( Children)	*Stylosanthes fruticosa* (Retz.) Alston	Leguminosae		Leaves	Herb	Oral	M
		Snake bite	*Stylosanthes fruticosa (*Retz.) Alston			Root		Oral	
75	Dolkoiso (M)	Stomach ache, Vomiting	*Tephrosia bracteolata* Guill. & Perr.	Leguminosae	291	Leaves	Shrub	Oral	M
76	Seringo demo golodo (M)	Gastritis	*Zornia pratensis* Milne-Redh.	Leguminosae	192, 328	Leaves	Herb	Oral	M
77	Polo Golodo (M)	Stomach ache, Diarrhea, Gastritis	*Abutilon longicuspe* Hochst. ex A.Rich	Malvaceae	193	Leaves	Herb	Oral	M
78	Puta (M)	Heart, food poisoning	*Gossypium herbaceum* L.	Malvaceae	621	Leaves	Shrub	Oral	M
		Ear ache	*Gossypium herbaceum* L.			Leaf bud		Topical	
79	Wari Beshe (M) Civil deshe (A)	Oral trash, Diarrhea , Malnutrition (Child)	*Kosteletzkya adoensis* (Hochst. ex A.Rich.) Mast.	Malvaceae	209,368,465	Leaves	Herb	Oral	M,
		Fresh cut to stop bleeding	*Kosteletzkya adoensis* (Hochst. ex A.Rich.) Mast.			Leaves	Herb	Topical	A1
80	Chuksha (A )	Swelling	*Sida rhombifolia* L.	Malvaceae	93, 314	Leaves	Herb	Topical	A2
81	Kautso (M)	Vomiting, Food poisoning	*Sterculia africana* (Lour.) Fiori	Malvaceae	47	Leaves	Tree	Oral	M
82	Gontersa (M)	Snake bite, Liver disease	*Bersama abyssinica* Fresen.	Melianthaceae	436,475	Stem bark	Shrub	Oral/ Topical	A1, A2
83	Chorahe (M)	Amoebiasis	*Chasmanthera dependens* Hochst.	Menispermaceae	100	Stem	Herb	Oral	M
		Swelling	*Chasmanthera dependens* Hochst.					Topical	
84	Balari (M)	Amoebiasis, Diarrhea, Rabies, Stomach ache	*Cissampelos mucronata* A.Rich*.*	Menispermaceae	29,96	Root	Herb	Oral	M
85	Haleko, Kellengi (A)	Eye disease (cataract)	*Moringa stenopetala (*Bak.) Cuf.	Moringaceae	-	Stem bark	Tree	Topical	M,
		Malaria	*Moringa stenopetala* (Bak.) Cuf*.*			Leaves	Tree	Oral	A1
86	Musi (A)	Diarrhea	*Musa paradisiaca* L.	Musaceae	-	Fruit	Herb	Oral	A1
87	Enkoko (A)	Tape worm	Embelia schimperi Vatke	Myrsinaceae	-	Seeds	Shrub	Oral	A2
88	Diko (M)	Lower extremity weakness, Rheumatism	*Commicarpus grandiflorus (*A.Rich) Standley	Nyctaginaceae		Leaves	Herb	Topical	M
89	Mukalle (M)	Wound	*Ximenia caffra* Sond.	Olacaceae	22	Seed	Tree	Topical	M
90	Chamo (M)	Tape worm	*Jasminum grandiflorum* L subsp. floribundum (R.Br. ex Fresen.) P.S.Green:	Oleaceae	415,727		Climber	Oral	M
91	Rimiti (M)	Ascariasis, Gonorrhea	*Olea europaea* L*. subsp. cuspidata* (Wall.ex G.Don) Cif.	Oleaceae	108	Leaves	Tree	Oral	M
92	Afi deshe (Bere Keno) (A)	Evil eye	*Biophytum umbraculum* Welw.	Oxalidaceae	264	Leaves	Herb	Oral	A2
93	Solcarindo (M), Kinsa kins (A)	Malnutrition (children) Diarrhea, Tooth ache, Stop fresh cut bleeding	*Oxalis radicosa* A. Rich.	Oxalidaceae	212, 326	Leaves with succulent stem	Herb	Oral	M, A1
94	Azi (ite) deshe	Swelling	*Phyllanthus ovalifolius* Forssk.	Phyllanthaceae	200	Leaves	Shrub	Topical	A1,A2
95	Afi Deshe (A)	Evil eye	*Phyllanthus rotundifolius* Willd	Phyllanthaceae	244, 305	Leaves	Herb	Oral	A1
96	Tolsi (M), Andod (A)	Gonorrhea, Stomach bloating	*Phytolacca dodecandra* L 'Hérit.	Phytolaccaceae	97,241	Leaves	Climber	Oral	M,A1
97	Kurupe (M), Guni deshe (A)	Tooth ache	*Plumbago zeylanica* L.	Plumbaginaceae	90, 279, 284,306,367	Root bark	Climber	Topical	M, A1,A2
		Snake bite	*Plumbago zeylanica* L.			Whole		Oral	
98	Tsoralle (M)	Skin burns	*Portulaca quadrifida* L*.*	Portulacaceae		Leaves	Herb	Topical	M
99	Wuchanbe (M)	Tape worm	*Myrsine africana* L.	Primulaceae	178	Seed	Shrub	Oral	M
100	Gero (M)	Tooth ache	*Faurea speciosa* Welw.	Proteaceae	109,413	Leaves	Tree	Topical	M
101	Dishoo (M)	Ear ache	*Clematis hirsuta* Perr. & Guill.	Ranunculaceae	110,231	Leaves	Climber	Topical	M
		Headache	*Clematis hirsuta* Perr. & Guill.			Leaves		Oral	
102	Afi Deshe (M)	Evil eye	*Ranunculus multifidus* Forssk.	Ranunculaceae	381,111, 270	Leaves	Herb	Topical	A2
		Tonsillitis	*Ranunculus multifidus* Forssk.					Oral	
103	Ziambee	Ascariasis	*Caylusea abyssinica* (Fresen.) Fisch. & Mey	Resedaceae	16	Leaves	Herb	Oral	M
104	Kulmi (A)	Tonsilities	*Rhamnus prinoides* L.'Hérit.	Rhamnaceae	-	Leaves	Shrub	Oral	A1,A2
105	Kosso (A)	Tape worm	*Hagenia abyssinica (*Brace) J.F.Gmel.	Rosaceae	-	Flower	Tree	Oral	M, A2
106	Afi Deshe(b)	Evil eye	*Oldenlandia lancifolia (*Schumach.) DC.	Rubiaceae	245	Leaves	Herb	Oral	A1
107	Wari ampi (M)	Liver disease (Ara)	*Pavetta gardeniifolia* A.Rich.	Rubiaceae	325	Leaves	Shrub	Inhale	M
		Common cold	*Pavetta gardeniifolia* A Rich.						
108	Afi deshe/gaina deshe (A)	Diarrhea, Evil eye, Tooth ache, Stomach ache, Head wound	*Pentas lanceolata* (Forssk.) Deflers	Rubiaceae	277,243,250, 276, 249	Root	Herb	Oral	A1, A2
109	Garo (M)	Ascariasis	*Vangueria apiculata* K. Schum.	Rubiaceae	8,157	Leaves	Shrub	Oral	M
110	Gembala (M)	Malaria	*Gardenia ternifolia* Schumach. & Thonn.	Rubiaceae	352	Leaves	Tree	Oral	M
111	Onaki (M)	Ascariasis	*Meyna tetraphylla* (Schweinf. ex Hiern) Robyns	Rubiaceae	4	Leaves	Tree	Oral	M
112	Lomi (A)	Oral trash; Food poisoning	*Citrus aurantiifolia* (Christm.) Swingle	Rutaceae	-	Fruit	Tree	Oral	A1
113	Tselto (M)	Stomach ache, common cold	*Ruta chalepensis* L. var.tenuifolia D'Urville	Rutaceae	-	Leaves with succulent stem	Herb	Oral	M, A1.A2
114	Gedai (M)	Common cold	*Zanthoxylum chalybeum* Engl.	Rutaceae	31	Seed	Tree	Oral	M
115	Wulchi (M)	Scorpion bite	*Anemia schimperiana* Presl.	Schizaeaceae	290	Leaves	Herb	Oral	M
116	Mitmita (A)	Malaria	*Capsicum annuum* L.	Solanaceae	-	Fruit	Herb	Oral	A1
117	Guni deshe (A)	Snake bite	*Datura metel* L.	Solanaceae	103	Whole	Herb	Oral/topical	A1
118	Atsefaris (M)	Toothache	*Datura stramonium* L*.*	Solanaceae		Leaf bud	Shrub	Topical	M
119	Ara Deshe (A)	Liver disease	*Discopodium penninervium* Hochst.	Solanaceae	339	Leaves	Tree	Inhale	A2
120	Tumbaho (M)	Leeches	*Nicotiana tabacum* L.	Solanaceae	-	Leaves	Herb	Topical/ Oral	A1,A2
121	Achi Kolpo (M), Garenti (A)	Amoebiasis, Stomach ache, Evil eye	*Solanum dasyphyllum* Schumach.	Solanaceae	9,94	Root	Herb	Oral	M,A1,A2
122	Kotse Garenti (A) Bulabulo(M)	Ascariasis, stomach ache	*Solanum incanum* L.	Solanaceae	94	Root	Herb	Oral	M
123	Muto (M)	Cold	*Withania somnifera* (L.) Dunal	Solanaceae	176, 208	Root	Herb	Oral	M
124	Azi deshe/masna (A)	Swelling	*Veronica abyssinica* Fres.	Scrophulariaceae	316, 334	Leaves	Herb	Topical	A2
125	Enaro (M)	Headache, Vomiting	*Lantana camara* L.	Verbenaceae	169	Leaves	Shrub	Oral	M
126	Dolo amede (M)	Headache	*Lantana trifolia* L.	Verbenaceae	190	Leaves	Shrub	Oral	M
127	Atuch	Stomach ache	*Verbena officinalis* subsp. *africana* R.Fernandes & Verdc.	Verbenaceae	268	Leaves	Herb	Oral	A2
128	Kuze (M)	Ascariasis, Food poisoning ,Vomiting	*Balanites rotundifolia (van Tieghem) Blatter*	Zygophyllaceae	41	Leaves	Tree	Oral	M

Study sites: M= Maale, A1= Ari 1and A2= Ari 2 ; Plant names are checked based on http://www.theplantlist.org.

Malaria, diarrhoea, ascariasis and amoebiasis were the most frequently cited health problems in Maale. Ascariasis is a helminthic human infection caused by *Ascaris lumbricoides*, a large roundworm. It is found worldwide with highest prevalence in tropical and subtropical regions, and in areas with inadequate sanitation [38,39]. Malaria, headache, stomach ache and diarrhoea were the main cited ailments in Ari 1 and Ari 2. Respondents said they identified and diagnosed the type of ailments by visual observation of the human body. Yellow, white of the eyes, for example, indicated liver health problem. Lulekal et al. [40] reported similar ways of diagnosis among traditional healers in Mana Angetu, southeastern Ethiopia. On the other hand, tape worm and ascariasis were diagnosed by observation of the worms in human faeces by the patients themselves or elders in the case of young children.

The average number of medicinal plants cited by each study participant of different age and gender groups is displayed in Table 2. The highest numbers of species were mentioned by participants from the Maale ethnic community. Moreover, in all study sites the results revealed that male participants mentioned a higher number of medicinal plants than female ones (t-test, p < 0.05). Our results are in agreement with the study results reported for the Bench ethnic communities in south-western Ethiopia by Giday et al. [16] that they found that the male study participants to have greater plant knowledge than females, because boys were favoured for the transfer of medicinal plant knowledge.

**Table 2:** Average number of medicinal plants cited per respondent groups

**Study sites**	**Gender**		**T-test**	**Age groups**		**T-test**
	**Male**	**Female**	**P-value**	**> 40**	≤**40**	**P-value**
Maale (n = 26 )	20.33	13.64	0.01*	19.67	12.73	0.01*
Ari 1(n = 24)	9.40	5.00	0.00*	8.06	7.22	0.65
Ari 2 (n = 24)	7.57	3.80	0.00*	6.77	5.09	0.13
Overall			0.01*			

People aged above 40 are considered matured adult by the community; P-values < 0.05 are marked with*.

In all study sites more medicinal plants were reported by participants over 40 years of age than by younger ones but this difference was significant for the Maale site. Awas [11] found that older people knew more than the youngsters in his study of the Kefficho people, in south-western Ethiopia. This may be due to the fact that knowledge tends to be accumulated through time. The relative lack of knowledge in the young will further be aggravated in the future when many species become scarce in the landscape and this might have negative impact on knowledge continuity in the near future. On the other hand, the results from Ari showed that knowledge is not always disappearing, as there were no significant differences in plant knowledge between age groups.

Table 3 shows the familiarity indices of medicinal plants for the treatment of different types of health problems. *Meyna tetraphylla* in Maale and *Solanum dasyphyllum* in Ari 1 and 2 were most cited. There was little correspondence between the two sites with regard to frequently mentioned plant species, but health problems treated with medicinal plants were quite similar. Species with high familiarity indices should be considered for further phytochemical and pharmacological studies.

**Table 3:** Familiarity index (FI) of medicinal plants In Maale (M), Ari 1 (A1) and Ari 2 (A2)

**Scientific name**	**Family name**	**Illness**	**Frequency**	**FI**	**Site**
*Meyna tetraphylla*	Rubiaceae	Ascariasis	11	0.42	M
*Plectranthus barbatus*	Lamiaceae	Amoebiasis	11	0.42	M
*Ozoroa insignis*	Anacardiaceae	Ascariasis	11	0.42	M
*Hypoestes forskaolii*	Acanthaceae	Stomach ache	10	0.38	M
*Ocimum basilicum*	Lamiaceae	Stomach ache	10	0.38	M
*Celosia trigyna*	Amaranthaceae	Tapeworm	10	0.38	M
*Plectranthus barbatus*	Lamiaceae	Stomach ache	10	0.38	M
*Solanum dasyphyllum*	Solanaceae	Stomach ache	16	0.67	A1
*Indigofera spicata*	Leguminosae	Ascariasis	12	0.50	A1
*Ruta chalepensis*	Rutaceae	Stomach ache	12	0.50	A1
*Plumbago zeylanica*	Plumbaginaceae	Snake bite	10	0.42	A1
*Acmella caulirhiza*	Compositae	Tonsillitis	9	0.38	A1
*Kosteletzkya adoensis*	Malvaceae	Fresh cut to stop bleeding	9	0.38	A1
*Vernonia amygdalina*	Compositae	Malaria	9	0.38	A1
*Citrus aurantifolia*	Rutaceae	Oral trash	9	0.38	A1
*Acmella caulirhiza*	Compositae	Tooth ache	8	0.33	A1
*Oxalis radicosa*	Oxalidaceae	Fresh cut to stop bleeding	8	0.33	A1
*Rhamnus prinoides*	Rhamnaceae	Tonsillitis	8	0.33	A1
*Citrus aurantifolia*	Rutaceae	Food poisoning	8	0.33	A1
*Solanum dasyphyllum*	Solanaceae	Stomach ache	10	0.42	A2
*Garcinia livingstonei*	Clusiaceae	Oral trash	10	0.42	A2
*Millettia ferruginea*	Leguminosae	Close wound caused by Jiggers	9	0.38	A2
*Nicotiana tabacum*	Solanaceae	Leeches	8	0.33	A2
*Hagenia abyssinica*	Rosaceae	Tape worm	8	0.33	A2

Preference ranking among ten key informants for eight selected medicinal plants used for the treatment of ascariasis is shown in Table 4. From Table 4 it appears that people had certain preferences for medicinal plants based on their perceived efficacy for the treatment of the frequently cited health problem, ascariasis. Species with higher preference ranking may indicate effective healing properties, which suggests that they are interesting for further phytochemical and pharmacological research.

**Table 4:** Preference ranking of eight medicinal plants used for the treatment of ascariasis based on perceived efficacy by ten respondents in Maale

**Scientific name**	**Total sum of ranks (n = 10)**	**Standard deviation**	**Mean ranking**	**Rank values**
*Ozoroa insignis*	18	± 1.0	1.8	1
*Meyna tetraphylla*	22	± 1.4	2.2	2
*Indigofera spicata*	34	± 1.6	3.4	3
*Vangueria apiculata*	45	± 1.6	4.5	4
*Balanites rotundifolia*	54	± *2.1*	*5.4*	*5*
*Pergularia daemia*	59	± *1.7*	*5.9*	*6*
*Senna singueana*	61	± *0.6*	*6.1*	*7*
*Combretum aculeatum*	67	±*2.0*	*6.7*	*8*

### Mechanisms of knowledge transfer among social groups

Most medicinal plant knowledge is transferred orally, as was reported by 71 study participants (96%) in the study sites (Table 5). This is the dominant mechanism of traditional knowledge transfer system in Africa [8], although this type of transfer cannot guarantee continuity under the current circumstances, where plant resource degradation and loss is severe. Most people (82%) obtained their knowledge from their (grand) parents, which is similar to the percentage found by a study in Wonago district, Ethiopia [41]. The great majority of the study participants preferred to transfer their medicinal plant knowledge to their children or grandchildren, which favours knowledge conservation and continuity mostly within the family line.

**Table 5:** Acquisition and willingness to transfer medicinal plant knowledge in the study sites

	**Maale frequency (%)**	**Ari 1 frequency (%)**	**Ari 2 frequency (%)**	**Total frequency (%)**
**Knowledge acquired**				
Parents/grandparents	20 (76.9)	19 (79.1)	22 (91.7)	61 (81.4)
Friends	1 (3.9)	1 (4.2)	0 (0.0)	2 (2.7)
Neighbours	2 (7.7)	1 (4.2)	0 (0.0)	3 (4.1)
Other (Given from God, accidentally encountered individuals)	3 (11.5)	3 (12.5)	2 (8.3)	8 (10.8)
**Knowledge willing to transfer to:**				
Children and grand children	17 (65.4)	22 (91.6)	20 (87.5)	59 (79.7)
Any family member	4 (15.4)	1 (4.2)	1 (4.2)	6 (8.1)
Neighbours	4(15.4)	0(0.0)	0 (0.0)	4 (5.4)
No one	1(3.8)	1(4.2)	3 (12.5)	5 (6.8)

The majority of study participants who showed interest to transfer their medicinal plant knowledge, preferred to transfer this to their first son. This preference was associated with the perception and fear that daughters would share the knowledge with their husbands’ family when they get married. Knowledge transfer to the new family was not appreciated by respondents with the perception that their secret knowledge would be known by others. When the first son was not considered trustworthy to keep the knowledge secretly or not judged interested in the subject as assessed through what he says, what he does and his general attitudes, parents transferred it to their second son or grandson.

In most cases there existed a concern among communities and elder knowledgeable people on plant resource degradation around their village. Dwindling resources around settlements may have negative implications for the future transfer of medicinal plant knowledge, as elders are unable to walk long distances from their residence. If the plants are no longer available, it becomes difficult to show and teach others about their names, characteristics and uses.

### Demographic factors influencing medicinal plant knowledge

Gender significantly predicted medicinal plant knowledge. Male study participants knew a higher number of medicinal plants than female ones. This is probably associated with the perception and culture of both ethnic communities to favour males in transferring medicinal plant knowledge. This must have implications for the cultivation of medicinal plant species in home gardens as women play a major role in managing these gardens. Moreover, site also strongly influenced the number of medicinal plants known and used (Table 6). Religion, family size and education did not influence plant knowledge.

**Table 6:** Socio-demographic and site factors prediction on the number of medicinal plant knowledge

	**Unstandardized**		**Standardized**	
	**Beta coefficient**	**Std. error**	**Beta coefficient**	**p-value**
R^2^ = 74.2				
Constant	3.831	0.443		
Age	0.016	0.006	0.198	0.014*
Gender	-0.819	0.149	-0.419	0.000*
Educational level	0.266	0.167	0.135	0.117
Family size	-0.041	0.025	-0.122	0.101
Site (Ari 1)	-1.485	0.185	-0.716	0.000*
Site (Ari 2)	-1.905	0.189	-0.918	0.000*
Religion (Protestant Christian)	0.136	0.178	0.068	0.449
Religion (Orthodox Christian)	0.190	0.199	0.093	0.343

Sites are compared against Maale site. Religion is compared against traditional religion. Variables with a significant influence (P<0.05) are marked with*.

Older members of the community in Maale knew more medicinal plants than youngsters (Table 2), which may also reflect an ongoing gradual knowledge loss of knowledge in the study community. Hence, it is important to include traditional knowledge in the school curricula to raise awareness as recommended by Awas [11].

### Similarities on in medicinal plant knowledge among sites

Generally we found little similarity among the three study sites. The calculated Jaccard similarity index was relatively higher between the two Ari sites (0.33), lower between Maale and Ari 1 (0.14) and lowest between Maale and Ari 2 (0.08). Our results showed that the Ari 1 and Ari 2 sites are more similar in medicinal plant knowledge than each of them compared to Maale. This can be explained by the geographical proximity between the two Ari sites and also supported by the fact that they belong to the same ethnic group and share their cultural background.

### Medicinal plant collection, conservation efforts and major threats

Study participants mostly collected medicinal plants from crop fields, home gardens and nearby forest patches. The results of the growth form analysis revealed that herbs were the most common growth form and the dominant plant parts harvested were leaves. This was found by many researchers in different parts of East Africa [42,43]. Harvesting of leaves may not have negative effects on resource availability, provided that the plant itself is not destroyed during harvesting, which is especially relevant for herbs.

A few study participants brought seedlings of medicinal herbs from fields and forest patches and started cultivating them in their home gardens. Their main reasons for doing so were to conserve plants that were scarce in their surroundings, to keep herbs available that were unavailable during the dry season and to have the medicine at hand during emergency situations. The practice of nurturing of wild species in home gardens was limited to a very small area, because people suspected that their importance as medicine could be easily guessed by outsiders. This unwillingness to share resources with neighbours or other non-family members may negatively affect the conservation of medicinal plant knowledge and resources. Giday and Teklehaymanot [44] did not encounter any cultivation of medicinal plants by Afar people in Ethiopian Ada’ar district. The main reason mentioned for this was the easily availability of the medicinal plant in areas that are not far from the homesteads.

Agricultural expansion and lack of cultivation tradition were indicated by our study participants as major threats in all study sites (Table 7). The conversion of natural vegetation to agricultural fields is a serious issue in Eastern Africa, where the rural population is highly dependent on subsistence agriculture [45]. Sustainable management of resources may not be an easy task, but it is crucial to guarantee future access to herbal medicine for rural communities [46]. Other threats that were mentioned were a lack of maintenance and the fact that people with little knowledge considered medicinal plants growing among their crops as weeds were likely to uproot them.

**Table 7:** Threats for medicinal plant resources and their priority ranking

**Threats**	**Herbs**			**Trees, shrubs, climbers**		
	**Maale**	**Ari 1**	**Ari 2**	**Maale**	**Ari 1**	**Ari 2**
Agricultural land expansion	2	2	2	1	1	1
Lack of cultivation and maintenance	1	1	1	2	2	2
Conflicting uses (timber, fencing, firewood, etc)	0	0	0	3	3	3
Grazing pressure	4	4	4	4	5	4
Drought or unreliable rainfall	3	3	3	5	4	5

“0”’ = not mentioned as a threat.

Most respondents (76% in Maale, 75% in Ari 1, 83% in Ari 2) collected plants at any time or day of the week. A few herbalists did not harvest plants on Sundays and some orthodox Christians did not harvest on Wednesdays and Fridays as these are fasting days. In emergency cases, they used stored herbal medicine on these days.

### Commercialization of herbal medicine

Marketing of medicinal plants was not common at the studied markets, apart from the well-known *Hagenia abyssinica* flowers and *Embelia shimperi* seeds (both wild collected) and the cultivated *Allium sativum* and *Artemisia absinthium* that are also used as spices. The commercialization of other wild and semi-wild species is hampered by the fact that medicinal knowledge is only held by few people. Tolassa [21] also found only a few species (*Thalictrum rhynchocarpum, Piper capense* and *Echinops kebericho*) at Gimibi and Gaba Senbeta markets, in western Ethiopia, while Giday et al. [16] found that the few species sold by Bench communities in south-western Ethiopia doubled as spices. In our study area, market chains were short and medicinal plants were directly sold by harvesters without further processing. The economic importance of the trade was limited: the price of *Embelia shimperii* seeds was only 2 Ethiopian Birr (0.10 $) per glass (about 250 ml). The product was not always available and marketed in small quantities.

Although herbalists’ incomes obtained through giving treatments to local communities were not high, the most important aspect observed from traditional healers is local recognition and respect by the community. In our study sites, respondents mentioned that on average they were consulted by patients five times per month. The charges for a treatment depended on the type of health problem treated and on patient/healers’ relationships. Payment per treatment ranged from 1-10 Ethiopian Birr (equivalent to 0.05 - 0.5 $) and sometimes were free of charge, especially in Maale area. However, in the Ari sites traditional healers believed that whatever relation existed, the patient had to pay money for a consult; otherwise they underlined that the medicine would not be effective. Limited income obtained from marketing of medicinal plants or from treatments given to patients may have negative implications future cultivation, maintenance and conservation of medicinal plants in the landscape.

## Conclusion

This study indicated that medicinal plants were important for the health care of the Maale and Ari communities, as they used at least 128 species and traditional medicine was considered as the first line of treatment by 89% of our respondents. Knowledge differed between and within ethnic groups and also among sites. The fact that knowledge transfer was predominantly to family members and in particular to first-born sons may negatively affect its continuity and may result in knowledge loss if medicinal plant resources become scarce in the future. Low income obtained from marketing of medicinal plants and herbal treatments may have strong implications on the future conservation of medicinal plants in the landscape.

Agricultural land expansion and a lack of cultivation practices limit the availability of medicinal plant resources in the area. Urgent action is required towards conservation (both ex-situ and in-situ combined) of medicinal plants and traditional knowledge before we lose them in the near future. Moreover, land use planning and development plan should also consider strategies that stimulate medicinal plant availability in the landscape and work towards increasing their cultivation to complement ex-and in-situ conservation efforts.

Popular medicinal species such as *Solanum dasyphyllum, Indigofera spicata, Plumbago zeylanica, Meyna tetraphylla* and multi-use species like *Oxalis radicosa* are good candidates for consideration in further phytochemical and pharmacological research to verify their efficacy.

## Competing interests

The authors declare that they have no competing interests.

## Authors’ contributions

The first author collected and analyzed the data, and wrote the draft manuscript and other authors made comments on the data analysis and on the write-up of the manuscript. All authors read and approved the final manuscript.

## References

[B1] Chaya N Poor access to health Services: Ways Ethiopia is overcoming it Res Comment 2007 2 2 1 6

[B2] IBC National biodiversity strategy and action plan 2005 Addis Ababa, Ethiopia: Government of the federal Democratic Republic of Ethiopia 103

[B3] Flatie T Gedif T Asres K Gebre-Mariam T Ethnomedical survey of Berta ethnic group Assosa Zone, Benishangul-Gumuz regional state, mid-west Ethiopia J Ethnobiol Ethnomed 2009 5 14 1 11 19409096 10.1186/1746-4269-5-14 PMC2688485

[B4] Gedif T Hahn HJ The use of medicinal plants in self-care in rural central Ethiopia J Ethnopharmacol 2003 87 155 161 10.1016/S0378-8741(03)00109-0 12860301

[B5] Yirga G Zeraburk S Ethnobotanical study of traditional medicinal plants of Gindeberet district, Western Ethiopia Mediterr J Soc Sci 2011 2 4 49 54

[B6] Joy PP Thomas J Mathew S Skaria BP Bose TK, Kabir J, Das P, Joy PP Medicinal Plants Trop Hort 2001 2 Calcutta: Naya Prokash 449 632

[B7] Bekele E Study on Actual Situation of Medicinal Plants in Ethiopia, Addis Ababa 2007

[B8] Fassil H We do what we know: local health knowledge and home-based medicinal plant use in Ethiopia 2003 PhD thesis Green College, Oxford University

[B9] Fassil H Beyond Plants, Professionals & Parchments: The role of home-based medicinal plant use and traditional health knowledge in primary health care in Ethiopia 2005

[B10] Giday M Medicinal plant of the Bench, Meinit and Sheko cultural groups in Ethiopia 2007 Addis Ababa, Ethiopia: PhD Thesis, Addis Ababa University

[B11] Awas T Plant diversity in Western Ethiopia Ecology, Ethnobotany and conservation 2007 Norway: PhD thesis, University of Oslo

[B12] Regassa T State constitutions in Federal Ethiopia: A preliminary observation: a summary for the Bellagio Conference 2004 1 12

[B13] Hunde D Asfaw Z Kelbessa E Use of traditional medicinal plants by people of ‘Boosat’ sub district, central eastern Ethiopia Ethiop J Health Sci 2006 16 2 141 155

[B14] Teklehaymanot T Giday M Ethnobotanical study of medicinal plants used by people in Zegie Peninsula, North-western Ethiopia J Ethnobiol Ethnomed 2007 3 12 1 11 17355645 10.1186/1746-4269-3-12 PMC1852296

[B15] Yineger H Yewhalaw D Teketay D Ethnomedicinal plant knowledge and practice of the Oromo ethnic group in south-western Ethiopia J Ethnobiol Ethnomed 2008 4 1 1 10 10.1186/1746-4269-4-1 18445249 PMC2390512

[B16] Giday M Asfaw Z Woldu Z Teklehaymanot T Medicinal plant knowledge of the Bench ethnic group of Ethiopia: an ethnobotanical investigation J Ethnobiol Ethnomed 2009 5 34 1 10 19912633 10.1186/1746-4269-5-34 PMC2780996

[B17] Teklehaymanot T Ethnobotanical study of knowledge and medicinal plants use by people in Dek Island in Ethiopia J Ethnopharmacol 2009 124 69 78 10.1016/j.jep.2009.04.005 19477609

[B18] Ashagre M Ethnobotanical Study of Medicinal Plants in Guji Agro-pastoralists 2011 Blue Hora District of Borana Zone: Oromia Region, Ethiopia. MSc thesis. Addis Ababa University

[B19] Dalle GT Maass BL Isselstein J Plant biodiversity and ethnobotany of pastoralists in southern Oromia Ethiop Econ Bot 2005 59 1 43 65 10.1663/0013-0001(2005)059[0043:PBAEOB]2.0.CO;2

[B20] Yirga G Assessment of indigenous knowledge of medicinal plants in Central zone of Tigray, Northern Ethiopia Afr J Plant Sci 2010 4 006 011

[B21] Tolasa E Use and conservation of traditional medicinal plants by indigenous people in Gimbi woreda, western Wellega, Ethiopia 2007 MSc thesis. Addis Ababa University 121

[B22] Friis BI Demissew S Van Breugel P Atlas of the Potential Vegetation of Ethiopia Biologiske Skrifter (Biol.Skr.Dan.Vid.Selsk.) 2010 58 1 307

[B23] CSA Summary and Statistical Report of the 2007 Population and Housing Censes 2008

[B24] Martin GJ Ethnobotany: a methods manual London 1995 UK: Chapman and Hall

[B25] Alexiades M Alexiades M Collecting ethnobotanical data. An introduction to basic concepts and techniques Selected Guideline for ethnobotanical research: A Field Manual 1996 U.S.A. Sheldon JW: The New York Botanical Garden 53 94

[B26] Cotton CM Ethnobotany: Principles and applications Chichester 1996 New York: John Wiley and Sons Ltd

[B27] Edwards S, Tadesse M, Hedberg I Canellaceae to Euphorbiaceous. Flora of Ethiopia and Eritrea Volume 2 1995 Addis Ababa, Ethiopia: Issue part 2. The National Herbarium Department of Systematic Botany, Uppsala, Sweden

[B28] Edwards S, Demissew S, Hedberg I Hydrocharitaceae to Arecaceae. Flora of Ethiopia and Eritrea Volume 6 1997 Addis Ababa, Ethiopia: The National Herbarium Department of Systematic Botany, Uppsala, Sweden

[B29] Edwards S, Tadesse M, Demissew S, Hedberg I Magnoliaceae to Flacourtiacea. Flora of Ethiopia and Eritrea Volume 2 Issue part 1 2000 Addis Ababa, Ethiopia: The National Herbarium Department of Systematic Botany, Uppsala, Sweden

[B30] Hedberg I, Edwards S Pittosporaceae to Araliaceae. Flora of Ethiopia Volume 3 1989 Addis Ababa, Ethiopia: The National Herbarium Department of Systematic Botany, Uppsala, Sweden

[B31] Hedberg I, Edwards S, Nemomissa S Apiaceae to Dipsacaceae. Flora of Ethiopia and Eritrea Volume 4 Issue part 1 2003 Addis Ababa, Ethiopia: The National Herbarium Department of Systematic Botany, Uppsala, Sweden

[B32] Hedberg I, Friis I, Edwards S Asteraceae Volume 4. Issue part 2. Flora of Ethiopia and Eritrea 2004 Addis Ababa, Ethiopia: The National Herbarium Department of Systematic Botany, Uppsala, Sweden

[B33] Hedberg I, Kelbesa E, Edwards S, Demissew S, Persson E Gentianaceae to Cyclocheilaceae Volume 5. Flora of Ethiopia and Eritrea 2006 Addis Ababa, Ethiopia: The National Herbarium Department of Systematic Botany, Uppsala, Sweden

[B34] The plant list a working list of all plant species 2013 (accessed date) http://Www.Theplantlist.Org. A working list of all plant species, Accessed date 2013

[B35] Höft M Barik SK Lykke AM Quantitative Ethnobotany. Applications of multivariate and statistical analyses in ethnobotany 1999 People and Plant Working Paper

[B36] Tabuti JRS Dhillion SS Lye KA The status of wild food plants in Bulamogi County, Uganda Int J Food Sci Nutr 2004 55 6 485 498 10.1080/09637480400015745 15762313

[B37] Pallant J SPSS survival manual, a step by step guide to data analysis using SPSS for Windows 2007 3

[B38] Ascariasis 2013 (accessed date) http://www.stanford.edu/class/humbio103/ParaSites2005/Ascaris/JLora_ParaSite.htm. Accessed date 2013

[B39] WHO Water Sanitation Health- Water related diseases 2013 WHO/WSH/ WWD/DFS.01

[B40] Lulekal E Kelbessa E Bekele T Yineger H An ethnobotanical study of medicinal plants in Mana Angetu District, south-eastern Ethiopia J Ethnobiol Ethnomed 2008 4 1 10 10.1186/1746-4269-4-1 18442379 PMC2391147

[B41] Mesfin F Demissew S Teklehaymanot T An ethnobotanical study of medicinal plants in Wonago Woreda, SNNPR Ethiopia J Ethnobiol Ethnomed 2009 5 28 1 18 19821994 10.1186/1746-4269-5-28 PMC2769162

[B42] Togola A Diallo D Dembele S Barsett H Paulsen BS Ethnopharmacological survey of different uses of seven medicinal plants from Mali, (West Africa) in the regions Doila, Kolokani and Siby J Ethnobiol Ethnomed 2005 1 7 1 9 10.1186/1746-4269-1-7 PMC1277087 16270940

[B43] Otieno NE Analo C Local indigenous knowledge about some medicinal plants in and around Kakamega forest in western Kenya F1000Res 2012 1 40 1 17 24701341 10.12688/f1000research.1-40.v1 PMC3954169

[B44] Giday M Teklehaymanot T Ethnobotanical study of plants used in management of livestock health problems by Afar people of Ada’ar District, Afar Regional State Ethiopia J Ethnobiol Ethnomed 2013 9 8 1 10 23343251 10.1186/1746-4269-9-8 PMC3561197

[B45] NCAPD Seeking solutions for traditional herbal medicine: Kenya develops a national policy 2008 Nairobi, Kenya: National Coordinating Agency for Population & Development Policy brief No.1

[B46] Cunningham AB African medicinal plants: Setting priorities at the interface between conservation and primary health care People Plants Working Paper 1993 1 1 50

